# Improvement of Jet Lag and Travel Fatigue Symptoms and Their Association with Prior International Travel Experience in Junior Athletes

**DOI:** 10.3390/sports12080220

**Published:** 2024-08-14

**Authors:** Yuka Tsukahara, Hiroshi Kamada, Suguru Torii, Fumihiro Yamasawa

**Affiliations:** 1 Department of Sports Medicine, Tokyo Women’s College of Physical Education, Kunitachi 1868668, Japan; 2 Medical Committee, Japan Association of Athletics Federations (JAAF), Tokyo 1600013, Japan; hkamada@md.tsukuba.ac.jp (H.K.); shunto@waseda.jp (S.T.); yamasawa-f@marubeni.com (F.Y.); 3 Department of Orthopaedic Surgery, University of Tsukuba, Tsukuba 3058575, Japan; 4 Faculty of Sport Sciences, Waseda University, Tokorozawa 3591192, Japan

**Keywords:** jet lag, athletics, travel fatigue

## Abstract

Jet lag and travel fatigue can occur after crossing multiple time zones in a short period of time, possibly affecting various aspects of an athlete’s behavior. However, there are few studies regarding this issue, particularly considering junior athletes. This study aimed to investigate and quantify the symptoms of jet lag and travel fatigue and the factors impacting these conditions. A survey was completed by 41 Japanese junior athletes (21 men and 20 women), competing at an international game in Finland, to assess their performance, sleeping habits, digestion, fatigue, and jet lag on the first day of arrival and on the opening day of the competition. Although athletes awoke less often during sleep on the opening day of the competition compared with the first day, sleep time, ease of falling asleep, and sleep quality decreased significantly. Prior experience traveling abroad for international competitions was positively associated with improvements regarding ease of falling asleep (coefficient = 2.22, *p* = 0.01), quality of sleep (coefficient = 2.16, *p* = 0.02), and alertness after waking up (coefficient = 1.85, *p* = 0.05) by the opening day of the competition when compared with the results for athletes who had no such prior experiences. Junior athletes experience symptoms of jet lag and travel fatigue that may persist until the day of competition, and prior experience traveling abroad may help in alleviating their symptoms.

## 1. Introduction

Many athletes travel internationally for training and competition [1,2]. Therefore, it is necessary for these athletes to adjust to jet lag and travel fatigue to maintain or improve their performance [3,4]. Jet lag is a circadian phase disruption that occurs after rapidly traveling across multiple time zones, particularly by air [5,6]. It is caused by the disruption of the circadian rhythm resulting from the new external environment; however, the symptoms are usually transient, and most resolve within a few days [5,6]. However, these symptoms may affect athletic performance, and they should be resolved as soon as possible once the athletes reach their destination [7]. Symptoms range from sleep disruption, gastrointestinal problems, loss of appetite, fatigue, and loss of concentration [6]. It is difficult to distinguish these symptoms from travel fatigue, which occurs regardless of the mode of transport, travel direction, or the number of time zones crossed, with non-specific symptoms arising from the hassles inherent in travel, including the disruption of daily routines and exposure to different environments [5]. In general, the more time zones crossed, the more time it takes to recover from jet lag. Jet lag occurring from eastward travel requires more time to resolve compared with that caused by westward travel because the body clock rhythm is >24 h; thus, it is more difficult to adjust to changes that shorten the day [8]. For westward travel, symptoms related to jet lag were reported to improve after three days [9]. Previous studies have examined the effects of jet lag on athletes, some of whom reported no significant performance decline following travel [3,10,11]. Conversely, Chapman et al. indicated that squat jump velocity decreased immediately and subsequently increased 48 h after traveling from Australia to Canada; however, further research is required, and most of these studies involved adult athletes [3,4,6]. Currently, an increasing number of teenage athletes travel internationally, as many competitions are held worldwide. For example, since 2010, the International Olympic Committee has organized the Youth Olympic Games, and the U20 World Championships are held every alternate year. Therefore, further information regarding the mechanisms for coping with jet lag and travel fatigue in junior athletes is warranted; however, available data regarding the symptoms of jet lag and travel fatigue in junior athletes are scarce [12]. In addition, the importance of sleep for athletes, which is known to be affected by international travel [13,14,15], has been reported. Furthermore, previous studies have also indicated that athletes participating in individual sports exhibit decreased sleep quality compared with those involved in team sports [16,17]. Cultural and environmental backgrounds can also affect sleep duration. A previous study reported that Japanese athletes recorded fewer sleep hours per day compared to their American counterparts [18]. Since sleep duration can potentially affect jet lag symptoms, those with chronic sleep deprivation may be more susceptible to jet lag [19].

Chronotypes are reported to influence the ability to cope with jet lag. Morning-types, characterized by early waking, morning alertness, and earlier bedtimes, generally have less difficulty adjusting to jet lag when traveling eastward compared to evening-types, who prefer later waking times, exhibit evening alertness, and report later bedtimes [8,20]. Athletes are reported to express a higher ratio of morning-types compared to that in the general population, and many have been reported to prefer training in the morning [21,22]. Research on Korean athletes demonstrated that morning-type athletes exhibit higher performance and better sleep quality compared to evening-types [23]. Additionally, traveling athletes are susceptible to social jet lag due to the disparity between rest-day and game-day schedules [24]. Social jet lag has been identified as a risk factor of injury, potentially impacting athletic performance [24].

The present study aimed to investigate and quantify the symptoms of jet lag and travel fatigue, particularly those related to sleep in junior athletes and their effect on the performance of the athletes. In addition, this study attempted to determine whether the athletes could cope with jet lag and travel fatigue before the competition. Considering that junior athletes have less experience with international travel, we also hypothesized that athletes with prior experience in international travel would exhibit better coping skills for sleep-related symptoms.

## 2. Materials and Methods

### 2.1. Study Participants and Flight Schedules

**Figure 1:**
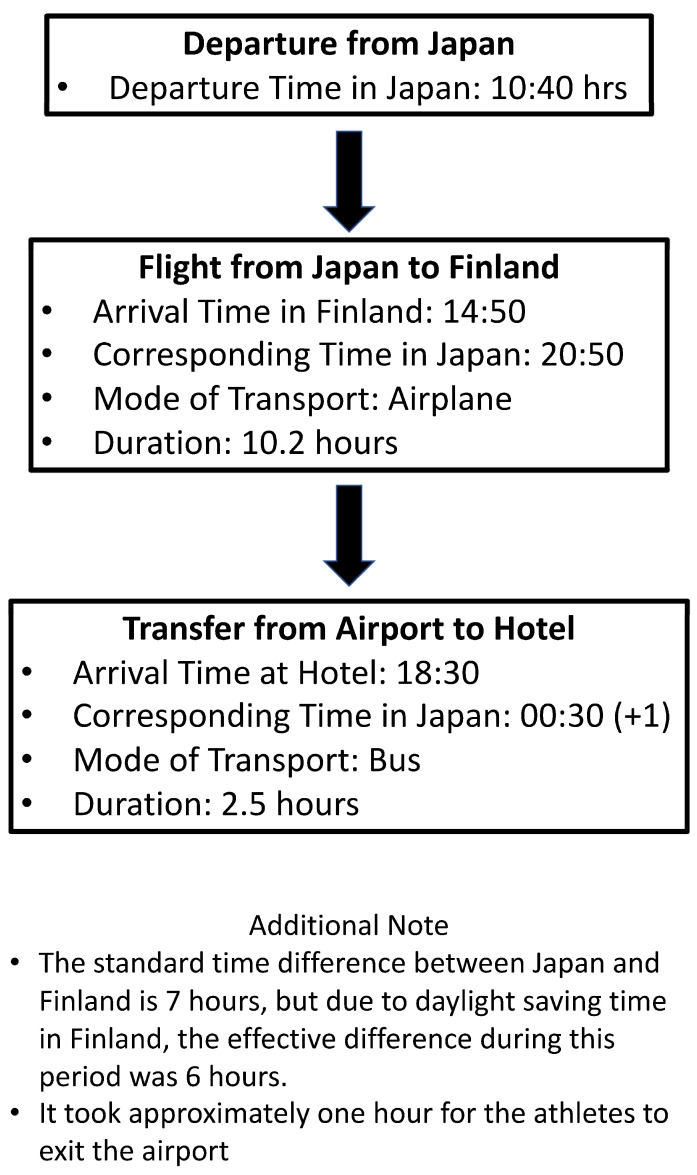
Athletes’ travel schedule.

In total, 46 athletes (26 men and 20 women) who competed in the 2018 World U20 Athletics Championships held in Finland were enrolled in this study. The study participants were those selected to compete in the 2018 World U20 Athletics Championships in July in Finland, which is 7 h behind Japan in terms of time zones. Written informed consent was obtained from all the participants, and the details of the study were communicated to their legal guardians. Since the flight departed early in the morning, all athletes stayed at the same hotel close by the airport. The athletes left the lobby at 8 a.m. and took a 10 min bus ride to the airport. The flight to Finland was a direct westward flight, departing Tokyo at 10:40 h and landing in Helsinki at 14:50 h. Subsequently, the athletes took a bus from Helsinki to Tampere. The flight time was 10.2 h, and the bus trip lasted 2.5 h. Details of their schedules are explained in Figure 1. After departing Tokyo, all the athletes traveled together, and they did not implement any specific countermeasures or sleep hygiene strategies before the flight. All athletes traveled in economy class, and they were not able to choose their own seats.

### 2.2. Survey

To ensure content validity, the survey underwent a thorough assessment by an expert committee comprising four sports medicine physicians with diverse backgrounds. The survey consisted of 16 questions, and it recorded the sex of the participants, their grade in school (grade 12, freshman, and sophomore; the ages are commonly 17–18, 18–19, and 19–20, respectively), and whether or not they had ever traveled internationally for athletics competitions. Participant characteristics, including height, body weight, and date of birth were not recorded, as the Japanese Olympic Committee has stopped announcing and publishing these data regarding elite athletes. Therefore, we respected this decision and did not collect this information. Previous studies have focused on chronotype and its association with jet lag via the administration of the Horne and Ostberg Morningness–Eveningness Questionnaire, which was not possible in this study, as some questions in the survey are related to work. Therefore, we simply asked the participants whether they considered themselves to be “morning-type,” meaning that they prefer to wake up early, or “evening-type,” meaning that they prefer to stay up late [25,26,27]. Except for sleep time and the frequency of interruptions during the night, which we asked them to record, the questions were to be answered at the end of each day using a visual analog scale (VAS). Although the Liverpool Jet Lag Questionnaire, which measures symptoms related to jet lag, has not been validated for the Japanese language, we referred to the questionnaire in our survey, and additional questions were revised to assist with the comprehension of junior athletes [28,29]. The VAS was set from −5 to 5, where −5 was the worst score, and 5 was the best. The survey was completed before bedtime: once on the day of arrival and again on the night before the competition, which was four days later. Details of the survey are listed in Figure S1.

### 2.3. Statistical Analysis

Wilcoxon test was performed to determine the difference between the scores for the night before game day (GD) and those for the arrival day (AD), which were 4 days apart. A paired t-test was used to compare the sleep hours. We subtracted the scores and calculated the difference between GD and AD to assess whether the symptoms were altered after arrival and to determine whether they became better or worse before the games. We performed a regression analysis to examine the related factors for the improvement of jet lag and travel fatigue. Independent factors included sex, chronotype, prior experience competing overseas for athletic games, and grade. All data were analyzed using Stata 16.1 (Stata Corporation, TX, USA). The significance level was *p* < 0.05. In addition, effect sizes based on Cohen’s d were determined for all variables to assist in the interpretation of any apparent trends. A small effect size (0.2 to 0.3) suggests a small but noticeable difference between groups; a medium effect size (0.5 to 0.7) reflects a moderate difference; and a large effect size (0.8 and above) indicates a substantial difference, with values exceeding 1 representing very large differences. Cronbach’s alpha was measured to evaluate the internal consistency of the survey. Data are presented as the mean ± standard deviation.

## 3. Results

Five male athletes failed to answer the survey and were excluded from the analysis. Thus, in total, 21 male and 20 female athletes were included in the study, of which 63.4% had previously experienced international travel for an athletics game, and 12.2% answered that they were “morning-type” individuals. None of the athletes were taking any substances, including medications and supplements that could interfere with sleep or performance. The majority of the respondents (61.0%) were freshmen in college. Details regarding the participants, including their chronotypes, are listed in Table 1.

Although self-reported performance and agility were not significantly different on AD vs. GD, flexibility decreased significantly on GD compared with that on AD (1.1 ± 1.2 for AD and 0.8 ± 1.3 for GD; *p* = 0.02). On GD, athletes had more difficulty falling asleep (3.3 ± 1.7 for AD and 2.2 ± 2.0 for GD; *p* = 0.02) and their quality of sleep (3.0 ± 1.5 for AD and 1.9 ± 1.9 for GD; *p* = 0.01) decreased significantly compared with that on AD. Sleep length and the number of times the athletes woke up during the night decreased significantly on GD compared with that on AD (sleep duration: 7.6 ± 0.8 h on GD and 8.3 ± 0.9 h for AD, respectively; nighttime awakenings: 1.1 ± 1.1 on GD and 0.2 ± 0.4 for AD, respectively). In addition, feeling hungry before meals (1.7 ± 1.5 for AD and 0.7 ± 1.2 for GD; *p* < 0.01) and feeling full after meals (0.9 ± 1.4 for AD and 0.3 ± 0.8 for GD; *p* = 0.02) decreased significantly on GD compared with that on AD. The results of the survey on AD vs. GD, along with the differences between the two, are listed in Table 2.

Factors related to sleep, including ease of falling asleep (coefficient 2.22, *p* = 0.01), quality of sleep (coefficient 2.16, *p* = 0.02), and alertness (coefficient 1.85, *p* = 0.05), improved significantly in athletes with international travel experience for athletics competition compared with those who had no prior experience. Male athletes woke up more often during their sleep, and the increase in the number of times they woke up during their stay was higher compared with that of their female counterparts (coefficient 1.03, *p* < 0.01). Sophomores had more difficulty falling asleep compared with their younger counterparts (coefficient −3.43, *p* < 0.01). The results of a linear regression analysis on sleep-related symptoms are listed in Table 3. The Cronbach’s alpha was 0.78 for AD and 0.81 for GD, respectively.

## 4. Discussion

This study focused on the symptoms related to jet lag and travel fatigue in elite junior athletes who traveled from Japan to Finland. To the best of our knowledge, this is the first study involving elite Japanese junior athletes. Most of the athletes answered that they were evening-type individuals, which is in agreement with the results of previous studies stating that adolescents tend to wake up at later times compared with adults, which may result from delayed circadian phases [30,31,32,33]. Overall, questions related to performance did not reveal any changes after a stay of 4 days. Although studies suggest that performance after traveling is lower compared with the baseline, this decrease in performance is limited after westward travel, which is consistent with our results, as the average of performance self-assessment was above zero, which indicates that most athletes did not have a negative assessment compared with normal results [4,34].

Wilcoxon’s analysis revealed that significant recovery was not observed after arrival. The only factor exhibiting significant improvement was the number of times the athletes awoke during sleep, which decreased before GD. Conversely, the number of hours of sleep decreased significantly. This may be explained by travel fatigue from the first day of travel and the ability to sleep long hours, but not because they were forced to wake up by jet lag, as the number of times they awoke during sleep decreased before the day of competition. This was similar to the results regarding the quality of sleep and the ease of getting to sleep. The quality of sleep decreased, and athletes found it significantly more difficult to fall asleep after their stay, probably because they were overly tired and experienced fewer problems getting to bed after traveling for half a day and sleeping in an aircraft, which causes sleep disturbances on AD[35]. Sex was not a factor related to most of the symptoms in the regression analysis, except in regards to the number of times the athletes woke up, which decreased more on GD compared with that on AD in female athletes than in their male counterparts. Cain et al. have reported that younger women have higher melatonin levels compared with men, which appears to be reflected in our data. However, further investigation including more participants is required [36]. Brown et al. also indicated the possibility that younger athletes may perform better according to their chronotype, and it has been acknowledged that morning-type athletes exhibit shorter endogenous periods compared with those of the evening-type participants [37]. If we follow this theory, since the athletes experienced westward travel, which is a phase delay, athletes with a shorter endogenous period should have more difficulty coping with jet lag. However, because it was not a related factor in any of the linear regression analyses, further studies, including validation using the Horne and Ostberg Morningness–Eveningness Questionnaire for athletes, is required [27].

Prior international travel experience for an athletics event was a positive factor associated with the ease of falling asleep, quality of sleep, and alertness. Because it was not possible to instruct the athletes on how to cope with jet lag before travel, it is speculated that those with experience had more knowledge from their previous trip(s) regarding how to cope with jet lag, indicating the importance, not only of experience, but also of education and prior knowledge. Furthermore, compared with high school athletes, sophomore athletes faced more difficulty falling asleep at 4 days post-arrival. Although it is difficult to arrive at a conclusion because the number of participants was small, previous studies have reported that older participants were more likely to be evening-type when compared to their younger counterparts [38]. Because the age difference is up to 2 years between 12th graders and sophomores, further studies involving more participants are required to determine the effect of age on sleep problems.

With respect to symptoms related to meals and digestion, feeling hungry before meals and full after meals decreased significantly after the athletes’ stay. The digestive circadian rhythm can shift after travel, as observed in our participants; however, most athletes reported that these symptoms were no worse than usual [39,40]. For symptoms related to the digestive system, it is hard to distinguish whether they were due to the disruption of the circadian rhythm, as appetite is also influenced by visual stimuli. If the athletes are eating a meal that is visually appealing or something they are familiar with, they will likely be satisfied with their meals and become hungry before meals [41,42]. Thus, these symptoms may depend on the food served to the athletes during their stay and the differences between these foods and those they are used to eating. Thus, further comparisons of different locations are necessary.

This study included several limitations. First, the symptoms of jet lag are thought to remain after returning to Japan; thus, we should also have evaluated symptoms both before and after travel to determine the baseline figures. Although Japan has only one time zone, this presented a difficulty, as the athletes lived in different parts of the country; thus, it would have been difficult to collect the data. Second, although many surveys related to jet lag exist, these have not been validated in Japanese, and particularly, for junior athletes. In the present study, although Cronbach’s alpha was sufficient, the test–retest reliability should have been measured alongside a baseline measurement. This baseline measure should involve conducting a survey before departure or using athletes who did not travel, ensuring that the results are not influenced by jet lag or travel fatigue.

Therefore, further studies are required to validate the surveys in Japanese and other languages. Furthermore, according to previous studies, the symptoms of jet lag depend on the time of the assessment, and because we only asked the subjects to fill out the questionnaire once a day before going to bed, it was not sufficient to conclude that the result may be an indicator of jet lag [7,43]. Thus, future studies should perform a validated survey multiple times per day, in addition to the baseline survey conducted before departure. However, asking athletes to fill out a questionnaire multiple times per day before a competition is challenging, and it is necessary to determine alternative effective methods for measuring jet lag and travel fatigue. Previous studies indicated that if a sport was played at a time when the core temperature was increased in the original time zone, the athletes could be in better condition for competing [39]. Therefore, scheduling is particularly important when traveling across several time zones. Furthermore, we were unable to investigate the subjective perception of recovery from jet lag and travel fatigue. Previous studies have used salivary cortisol levels, heart rates, and questionnaires to assess athletes’ recovery [44,45,46,47]. Therefore, monitoring physiological, hormonal, and psychometric outcomes that could potentially impact the athletes’ recovery status would have provided a more comprehensive assessment.

This study provides valuable insights into the impact of jet lag and travel fatigue on elite junior athletes traveling from Japan to Finland. Despite the limitations, such as the small sample size and the challenges in data collection across different time zones, our findings highlight significant aspects of how these athletes cope with long-distance travel. A decrease in flexibility after four days and a notable reduction in sleep quality and ease of falling asleep were observed. Interestingly, athletes with previous international travel experience showed better adaptation, suggesting the importance of educating young athletes on coping mechanisms for jet lag and travel fatigue. Additionally, this study underscores the need for more robust methods, such as actigraphy, for monitoring sleep, as well as further research to validate the surveys used. Future research should also explore the role of dietary factors and different travel destinations to provide a comprehensive understanding of the effects of jet lag and travel fatigue. Ultimately, equipping young athletes with the knowledge and tools to manage jet lag and travel fatigue will be crucial for optimizing their performance in international competitions. By doing so, and by understanding how to cope with jet lag and travel fatigue, young junior athletes can be better prepared to maintain their performance and progress to become elite athletes as they grow older.

## 5. Conclusions

Regardless of age, elite athletes are required to travel overseas for competitions and trainings, exposing them to jet lag and travel fatigue. These physiological effects may affect their performance. Thus, athletes need to be aware of what they can do to minimize the effects of jet lag and travel fatigue. Based on our results, athletes with prior international travel experience faced less difficulty coping with symptoms related to sleep, possibly due to their familiarity and know-how regarding travel fatigue issues. Thus, elite athletes competing at a young age should be provided with opportunities to understand how to minimize the effects of jet lag and travel fatigue. The findings of this study underscore the necessity for ongoing education and support systems for athletes to enable them to better manage travel-induced physiological stress. As international competitions become increasingly frequent, equipping athletes with comprehensive strategies to handle jet lag and travel fatigue will be essential in helping them to maintain their competitive edge. Further studies could explore the use of technology and validated surveys to monitor sleep patterns and other physiological parameters, providing a more detailed understanding of how to support athletes in different competitive environments.

## Figures and Tables

**Table 1:** Characteristics of the participants.

	Male (N = 21)	Female (N = 20)
**Chronotype**		
Morning type	2 (9.5%)	3 (15.0%)
Evening type	19 (90.5%)	17 (85.0%)
**Grade**		
Grade 12	3 (14.3%)	6 (30.0%)
Freshman	14 (66.7%)	11 (55.0%)
Sophomore	4 (19.0%)	3 (15.0%)
**Have previously traveled overseas for competitions**	10 (47.6%)	16 (80.0%)

**Table 2:** Results of the survey on arrival day and game day, along with the differences between them.

	Arrival Day	Game Day	*p* Value	Effect Size
Performance	1.3 ± 1.2	1.8 ± 1.7	0.41	−0.34
Agility	1.7 ± 1.3	1.7 ± 1.5	0.82	−0.02
Flexibility	1.1 ± 1.2	0.8 ± 1.3	0.02	0.28
Concentration	1.6 ± 1.4	2.1 ± 1.7	0.21	−0.35
Motivation	2.1 ± 1.7	2.6 ± 1.7	0.16	−0.31
Mood	1.5 ± 1.7	1.3 ± 1.6	0.71	0.12
Ease of getting to sleep	3.3 ± 1.7	2.2 ± 2.0	0.02	0.57
Quality of sleep	3.0 ± 1.5	1.9 ± 1.9	0.01	0.62
Alertness after waking	2.6 ± 1.7	2.0 ± 2.0	0.06	0.34
Sleep length (hours)	8.3 ± 0.9	7.6 ± 0.8	<0.01	0.78
Number of times waking at night (times)	1.1 ± 1.1	0.2 ± 0.4	<0.01	1.09
Hunger before meals	1.7 ± 1.5	0.7 ± 1.2	<0.01	0.73
Satisfaction after meals	1.8 ± 1.8	1.7 ± 2.0	0.55	0.05
Feeling full after meals	0.9 ± 1.4	0.3 ± 0.8	0.02	0.52
Bowel activity	0.7 ± 1.2	0.4 ± 1.2	0.10	0.20
Heavy stomach	0.8 ± 1.6	0.3 ± 0.9	0.91	0.34
Fatigue	1.5 ± 1.4	1.0 ± 1.1	0.06	0.41
Feeling jet lagged	2.1 ± 1.8	2.5 ± 2.3	0.85	−0.22

**Table 3:** Results of a linear regression analysis for factors related to changes in symptoms regarding sleep.

	Coefficient	95% CI	*p* Value
**Ease of getting to sleep**			
Sex (Ref: Female)			
Male	0.06	−1.51, 1.63	0.94
Chronological type (Ref: Morning type)			
Evening type	−2.07	−4.33, 0.18	0.07
Have previously traveled overseas for competitions (Ref: No)			
Yes	2.22	0.49, 3.95	**0.01**
Grade (Ref: High school 3rd year)			
Freshmen	−1.33	−3.16, 0.50	0.15
Sophomore	−3.43	−6.09, −0.77	**0.01**
**Quality of sleep**			
Sex (Ref: Female)			
Male	0.45	−1.17, 2.06	0.58
Chronological type (Ref: Morning type)			
Evening type	0.11	−2.21, 2.42	0.93
Have previously traveled overseas for competitions (Ref: No)			
Yes	2.16	0.38, 3.93	**0.02**
Grade (Ref: High school 3rd year)			
Freshmen	−0.46	−2.34, 1.42	0.62
Sophomore	−2.16	−4.89, 0.57	0.12
**Alertness after waking**			
Sex (Ref: Female)			
Male	−0.25	−1.93, 1.44	0.77
Chronological type (Ref: Morning type)			
Evening type	0.25	−2.16, 2.66	0.83
Have previuosly traveled overseas for competitions (Ref: No)			
Yes	1.85	0.01, 3.70	**0.05**
Grade (Ref: High school 3rd year)			
Freshmen	0.70	−1.26, 2.66	0.47
Sophomore	−0.86	−3.70, 1.99	0.55
**Sleep length**			
Sex (Ref: Female)			
Male	0.28	−1.08, 1.64	0.68
Chronological type (Ref: Morning type)			
Evening type	−0.92	−2.86, 1.03	0.35
Have previously traveled overseas for competitions (Ref: No)			
Yes	0.07	−1.42, 1.55	0.93
Grade (Ref: High school 3rd year)			
Freshmen	−1.43	−3.01, 0.15	0.07
Sophomore	−1.21	−3.50, 1.09	0.29
**Number of times woken up at night**			
Sex (Ref: Female)			
Male	1.03	0.38, 1.68	**<0.01**
Chronological type (Ref: Morning type)			
Evening type	0.38	−0.55, 1.31	0.41
Have previoulsy traveled overseas for competitions (Ref: No)			
Yes	−0.19	−0.90, 0.53	0.60
Grade (Ref: High school 3rd year)			
Freshmen	−0.30	−1.06, 0.45	0.42
Sophomore	−0.26	−1.35, 0.84	0.64

Bold values denote statistical significance at the *p* < 0.05 level.

## Data Availability

The data presented in this study are available on request from the corresponding author.

## Supplementary Materials

The following supporting information can be downloaded at: https://www.mdpi.com/article/10.3390/sports12080220/s1, Figure S1: Survey distributed to athletes.
