# Supplementary figures and images for: Improvement of Jet Lag and Travel Fatigue Symptoms and Their Association with Prior International Travel Experience in Junior Athletes
Source: Sports (Basel). 2024 Aug 14;12(8):220. doi: 10.3390/sports12080220 (PMC11358972; doi:10.3390/sports12080220)

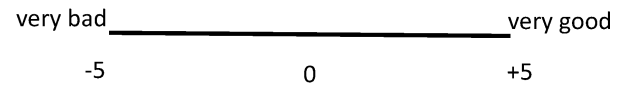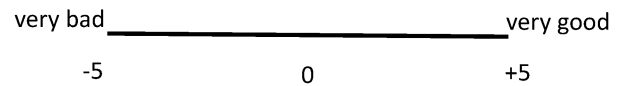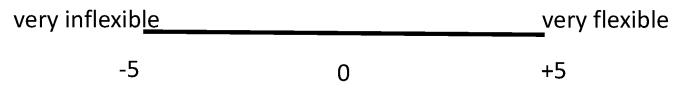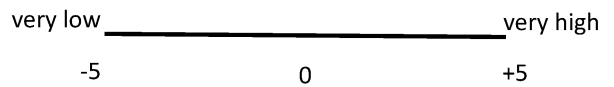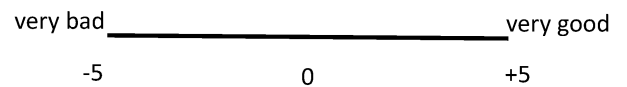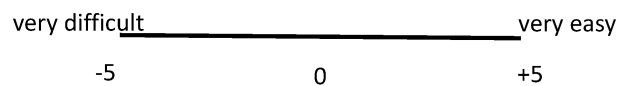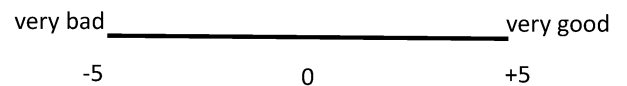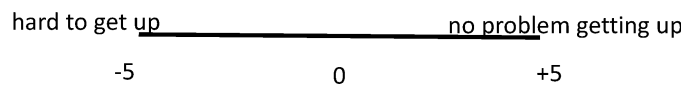

-5                      0                      +5

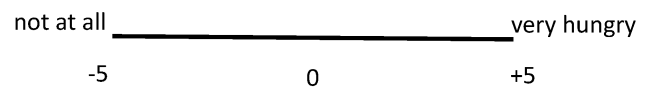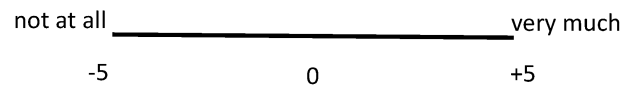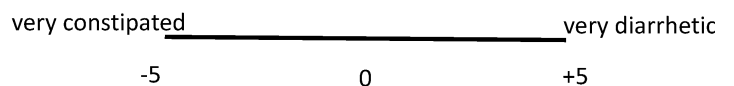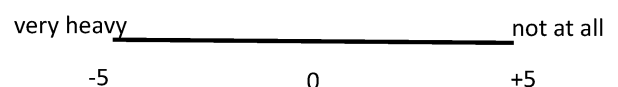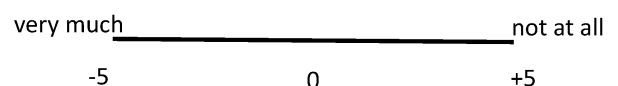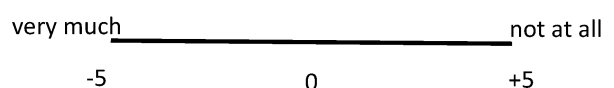

Supplement: Supplementary file 1 [file sports-12-00220-s001.zip › sports-3086105-Figure S1 Survey distributed to athletes.pdf]
